# Supplementary material for: Empirical methods for controlling false positives and estimating confidence in ChIP-Seq peaks
Source: BMC Bioinformatics. 2008 Dec 5;9:523. doi: 10.1186/1471-2105-9-523 (PMC2628906; doi:10.1186/1471-2105-9-523)
Supplement: Additional File 1 — A variety of html documents from the USeq web site detailing the available applications, their best usage, output file type descriptions, command line menus, etc. [file 1471-2105-9-523-S1.zip › USeqUserGuides/JavaDoc.zip!/JavaDoc/trans/main/T2.html]

T2


|  |  |  |
| --- | --- | --- |
| **Overview** \| **Package** \| **Class** \| **Use** \| **Tree** \| **Deprecated** \| **Index** \| **Help** | |  |
| **PREV CLASS**   **NEXT CLASS** | **FRAMES**    **NO FRAMES**     **All Classes** |  |
| SUMMARY: NESTED \| FIELD \| CONSTR \| METHOD | DETAIL: FIELD \| CONSTR \| METHOD |  |


---


## trans.main Class T2

```
java.lang.Object
  trans.main.T2
```

---

``` public class T2 extends java.lang.Object ```

Runs entire TiMAT2 suite based on a parameter file.

---

**Constructor Summary**

`T2(java.lang.String[] args)`


| **Method Summary** | |
| --- | --- |
| `void` | `convertCelFiles()` |
| `void` | `deleteSplitNormalizedFiles()` |
| `void` | `filterIntervals()`             Creates set number of intervals. |
| `java.lang.String[]` | `findChromPairs()`             Attempts to join smallest chromosomes together so long as the combine file doesn't exceed the size of the biggest. |
| `void` | `findSubBindingRegions()`             Creates set number of intervals. |
| `void` | `launchCelNormalization(java.io.File[] celaFiles, java.util.ArrayList generalCmd, java.io.File clusterName)` |
| `void` | `launchJQSub(java.util.ArrayList args, int memory)` |
| `void` | `loadIntervalArrays()`             Creates set number of intervals. |
| `static void` | `main(java.lang.String[] args)` |
| `void` | `makeAndMoveIntervals()`             File management for intervals. |
| `void` | `makeChromosomeSets()` |
| `void` | `makeChromSet(java.io.File[][] setRep, boolean treatmentSet)` |
| `void` | `mergeWindows()`             Creats one or two window merged window arrays containing all of the chromosomes. |
| `void` | `normalizeCelFiles()` |
| `int` | `numberOfIntervalsToMake()`             Returns the number of intervals that remain to be made. |
| `int` | `numberReplicasToMake()` |
| `static void` | `printDocs()` |
| `void` | `printIntervalGraphs()`             Prints interval bed and sgr files via IntervalGraphPrinter. |
| `void` | `printSpreadSheets()`             Prints interval reports in a spread sheet via IntervalReportPrinter. |
| `void` | `processArgs(java.lang.String[] args)`             This method will process each argument and assign new varibles |
| `void` | `scanChromosomes()` |
| `void` | `setNumberIntervalMaker()`             Creates set number of intervals. |
| `void` | `splitChromSetArray()`             Pulls and places replica directories to this. |

**Methods inherited from class java.lang.Object**

`equals, getClass, hashCode, notify, notifyAll, toString, wait, wait, wait`

**Constructor Detail**

### T2

```
public T2(java.lang.String[] args)
```


**Method Detail**

### numberOfIntervalsToMake

```
public int numberOfIntervalsToMake()
```

:   Returns the number of intervals that remain to be made.

---


### makeAndMoveIntervals

```
public void makeAndMoveIntervals()
```

:   File management for intervals.

---


### setNumberIntervalMaker

```
public void setNumberIntervalMaker()
```

:   Creates set number of intervals.

---


### loadIntervalArrays

```
public void loadIntervalArrays()
```

:   Creates set number of intervals.

---


### findSubBindingRegions

```
public void findSubBindingRegions()
```

:   Creates set number of intervals.

---


### printSpreadSheets

```
public void printSpreadSheets()
```

:   Prints interval reports in a spread sheet via IntervalReportPrinter.

---


### printIntervalGraphs

```
public void printIntervalGraphs()
```

:   Prints interval bed and sgr files via IntervalGraphPrinter.

---


### filterIntervals

```
public void filterIntervals()
```

:   Creates set number of intervals.

---


### mergeWindows

```
public void mergeWindows()
```

:   Creats one or two window merged window arrays containing all of the chromosomes.

---


### splitChromSetArray

```
public void splitChromSetArray()
```

:   Pulls and places replica directories to this.

---


### scanChromosomes

```
public void scanChromosomes()
```

---


### findChromPairs

```
public java.lang.String[] findChromPairs()
```

:   Attempts to join smallest chromosomes together so long as the combine file doesn't exceed the size of the biggest.

---


### deleteSplitNormalizedFiles

```
public void deleteSplitNormalizedFiles()
```

---


### makeChromSet

```
public void makeChromSet(java.io.File[][] setRep,
                         boolean treatmentSet)
```

---


### numberReplicasToMake

```
public int numberReplicasToMake()
```

---


### makeChromosomeSets

```
public void makeChromosomeSets()
```

---


### normalizeCelFiles

```
public void normalizeCelFiles()
```

---


### launchCelNormalization

```
public void launchCelNormalization(java.io.File[] celaFiles,
                                   java.util.ArrayList generalCmd,
                                   java.io.File clusterName)
```

---


### convertCelFiles

```
public void convertCelFiles()
```

---


### launchJQSub

```
public void launchJQSub(java.util.ArrayList args,
                        int memory)
```

---


### main

```
public static void main(java.lang.String[] args)
```

---


### processArgs

```
public void processArgs(java.lang.String[] args)
```

:   This method will process each argument and assign new varibles

---


### printDocs

```
public static void printDocs()
```


---


|  |  |  |
| --- | --- | --- |
| **Overview** \| **Package** \| **Class** \| **Use** \| **Tree** \| **Deprecated** \| **Index** \| **Help** | |  |
| **PREV CLASS**   **NEXT CLASS** | **FRAMES**    **NO FRAMES**     **All Classes** |  |
| SUMMARY: NESTED \| FIELD \| CONSTR \| METHOD | DETAIL: FIELD \| CONSTR \| METHOD |  |


---
